# Eutectic mixture of local anaesthetics for pain reduction during extracorporeal shockwave lithotripsy: A systematic review and meta-analysis

**DOI:** 10.1371/journal.pone.0237783

**Published:** 2020-10-05

**Authors:** Zhenghao Wang, Guo Chen, Jia Wang, Wuran Wei

**Affiliations:** Department of Urology, Institute of Urology, West China Hospital, Sichuan University, Chengdu, China; University of Aberdeen Institute of Applied Health Sciences, UNITED KINGDOM

## Abstract

A systematic review and meta-analysis was conducted to explore the effect of a eutectic mixture of local anaesthetics (EMLA) on pain reduction during extracorporeal shockwave lithotripsy (ESWL). PubMed, Web of Science, Embase, EBSCO, and Cochrane library databases (updated March 2020) were searched for randomised controlled trials (RCTs) assessing the effect of EMLA for patients that underwent ESWL. The search strategy and study selection process were managed according to the PRISMA statement. Six RCTs were included in the meta-analysis. Overall, the results indicated that EMLA significantly reduced pain compared to the control group (RR = -2.98, 95% CI = -5.82 to -0.13, P = 0.04) with a heterogeneity of I^2^ = 57% (P = 0.04). Subgroup analysis showed that EMLA did not significantly reduce pain when the patients took an analgesic premedication (RR = -1.46, 95% CI = -5.89 to 2.98, P = 0.52) with a heterogeneity of I^2^ = 38% (P = 0.52). Conversely, studies without premedication showed a significant pain relief effect (RR = -4.08, 95% CI = -7.36 to -0.65, P = -0.80) with a heterogeneity of I^2^ = 48% (P = 0.14). Most studies showed there was no difference in the patient’s need for analgesics. EMLA was effective for reducing pain during EWSL. However, this analgesic effect was limited and did not reduce the need for analgesics.

## Introduction

Urinary stone disease is the third common disease in the urinary tract which affects 1–5% of the population in Asia, 5–9% of the population in Europe, and 13% of the population in North America [1]. Patients with nephrolithiasis often suffer from various short- and long-term complications. Technological advances are continuously influencing the treatment patterns for urinary stones, which have shifted to less invasive procedures. As a result, extracorporeal shockwave lithotripsy (ESWL) has lost its place as mostly common therapy for renal and ureteral stones [2].

The advantages ESWL are that it is minimally invasive, lacks severe side effects, does not require the use of general anaesthesia, and is economical. Therefore, it has gained widespread acceptance and use for treating uncomplicated stones in certain areas of the kidney. However, the issue that affects patients the most is pain and anxiety after ESWL sessions. According to previous studies, pain may be the result of two factors. The primary source is the increased pressure within the kidney and the other is due to the ESWL-related trauma of the skin and muscles and the stretching of the surrounding renal capsule [3, 4]. Furthermore, the patient’s tolerance and the effectiveness of this procedure is strongly affected by both ESWL-related pain and anxiety [5]. To reduce the level of pain and anxiety and increase the compliance of the patients, various complementary procedures such as general anaesthesia and analgesia have been introduced [6]. Though these approaches have proven effective, it is not highly recommended due to the drug side-effects and costs [7].

Eutectic mixture of local anaesthetics (EMLA, 2.5% lidocaine and 2.5% prilocaine) is commonly used as a local anaesthetic topical cream. This cream penetrates the skin up to a depth of 4 mm and the onset time is 10–20 minutes and provides pain relief for up to 60 minutes [8]. Its effectiveness has been proven in many fields like skin analgesia, cannulation, and venepuncture [9]. Several comparative studies have reported the effect of EMLA on reducing pain and need for anaesthetic during ESWL. However, the results remain controversial. Therefore, we conducted a systematic review and meta-analysis of randomised controlled trials (RCTs) to explore the effectiveness of EMLA during ESWL.

## Method and materials

### Literature search and selection criteria

We systematically searched PubMed, Embase, Web of Science, EBSCO, and the Cochrane library up to March 2020 with the following keywords: “topical anaesthetics”, “eutectic mixture of local anaesthetics”, “pain”, and “shock wave lithotripsy”. The list of retrieved studies and relevant reviews were assessed manually and the process was repeated several times to ensure that all eligible studies were included. All processes were in accordance with the Cochrane Handbook [10]. Inclusion criteria were as follows: (1) RCT study design, (2) comparison between ESWL with EMLA versus ESWL with placebo, (3) adequate reporting of data provided for analysis, and (4) full text in English.

### Data extraction and outcome measures

Baseline information was extracted from the original studies and included the following: first author, published year, number of patients, patient age and gender distributions, description of calculus, and specific usage of EMLA. Data were independently extracted by two investigators. Discrepancies were resolved by consensus.

### Quality assessment of individual studies

The methodological quality of each RCT was assessed according to the Jadad scale, which comprises of the following three evaluation elements: randomisation (0–2 points), blinding (0–2 points), and dropouts and withdrawals (0–1 points) [11]. One point was awarded for each element that was conducted and appropriately described in the original article. The total score ranged from 0 to 5 points. An article with a Jadad score of ≤ 2 was considered to be of low quality while a Jadad score of ≥ 3 indicated a high-quality study [12].

### Statistical analysis

Risk ratio (RR) with 95% confidence interval (CI) was calculated for dichotomous outcomes. Study heterogeneity was evaluated using the I^2^ statistic with I^2^ > 50% taken to indicate significant heterogeneity [10]. Sensitivity analysis was performed to evaluate the influence of a single study on the overall outcome by omitting one study in turn or performing subgroup analyses. The random-effects model was used for meta-analysis. Owing to the limited number of included studies (< 10), publication bias was not assessed. Statistical significance was accepted at P < 0.05. All statistical analyses were performed using Review Manager Software Version 5.3 (The Cochrane Collaboration, UK).

## Results

### Literature search, study characteristics, and quality assessment

**Fig 1:**
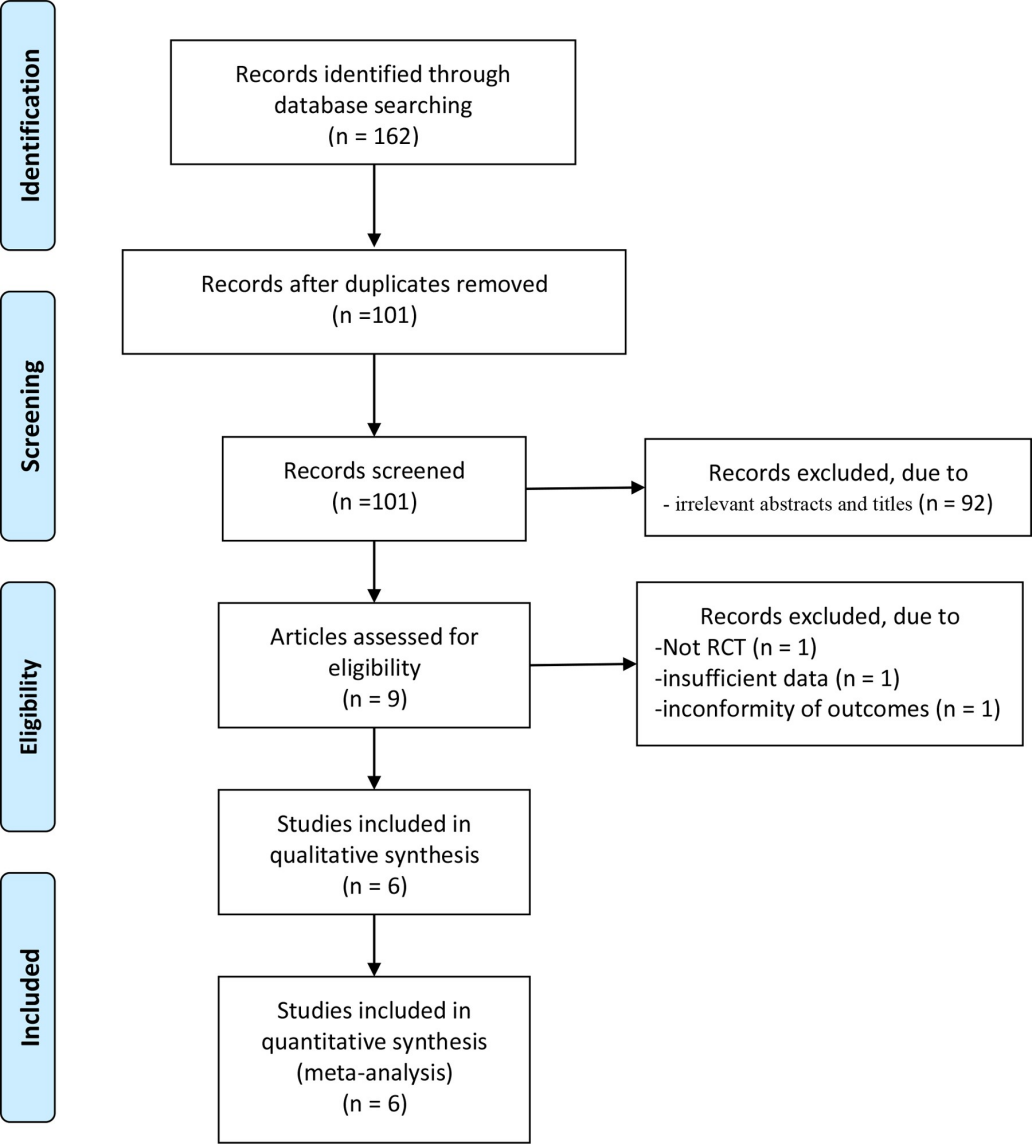
Flow diagram of study searching and selection process.

In total, 162 articles were initially identified from the databases. After removing duplicates, 101 articles were retained. Then 92 studies were excluded from our study due to unrelated abstracts and titles. We also excluded from our analysis: one article for its study design (not RCT), one articles for insufficient data, and one article for inconformity of outcomes. Finally, six RCTs that satisfied the inclusion criteria were enrolled in this meta-analysis [9, 13–17]. The article selection process followed the PRISMA statement (Fig 1). The baseline characteristics of the six included studies are shown in Table 1. The studies in our meta-analysis were published between 1993 and 2013 and the total sample size was 949. There was no significant difference in the baseline parameters and all studies demonstrated the effectiveness of EMLA. Premedication prior to ESWL was given in three studies. In the study of Tiselus *et al*., patient received an intramuscular injection of 75 mg meperidine hydrochloride and an oral dose of 5 mg diazepam as premedication 30 minutes before ESWL. Meperidine and diazepam were given intravenously if patients still reported pain during procedure [15]. Monk *et al*. administered 2 mg midazolam I.V to all patients before ESWL then provided intraoperative alfentanil during ESWL when required to further suppress discomfort [13]. The patients in Ganapathy *et al*. received either metoclopramide 10 mg or droperidol 1 mg as premedication and were then provided with alfentanil during the procedure [14]. The remaining studies did not use premedication. Acar *et al*. used remifentanil as the intraoperative analgesics [9]. Finally, Vilar *et al*. and Tritrakarn *et al*. did not supply their patients with any pain medication [16, 17]. Jadad scores of the included studies ranged from 4 to 5, thus were regarded as high-quality RCTs.

**Table 1:** Characteristics of included studies.

No.	Author	year	Experimental group					Control group					
			Number (n)	Age (Mean±SD)	Male (n)	Calculus location (Renal /Ureteral)	How to use the EMLA	Number (n)	Age (Mean±SD)	Male (n)	Calculus location (Renal /Ureteral)	Placebo	Jadad score
1	Tiselus	1993	99	-	66	67/32	30 g on 15*15cm skin covered by a plastic occlusive dressing at least 60 min	100	-	68	63/37	Placebo cream	4
2	Monk	1994	30	53±13	19	30/0	30 g on 15*20cm skin covered by a plastic occlusive dressing for 90 min	29	50±13	19	19/0	Placebo cream	5
3	Ganapathy	1996	44	47±12	32	44/0	30 g on 15*20cm skin covered by a plastic occlusive dressing for 60–90 min	39	47±12	25	39/0	Placebo cream	4
4	Tritrakarn	2000	78	40±10	50	78/0	10 g covered by a plastic occlusive dressing for 60 min, 39 patients remove the dressing before procedure	124	38±11	75	124/0	Placebo cream or no intervention	4
5	Vilar*	2012	165	47±16	98	123/42	Covering 10 cm^2^ area for 60 min	269	43±17	189	204/65	Placebo cream	4
6	Acar	2013	30	49±2	17	27/3	10g on 10*15cm skin	30	43±3	16	26/4	Placebo cream	4

### Pain control

**Fig 2:**
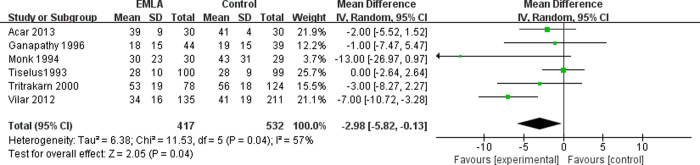
Forest plot for the meta-analysis of pain control.

**Fig 3:**
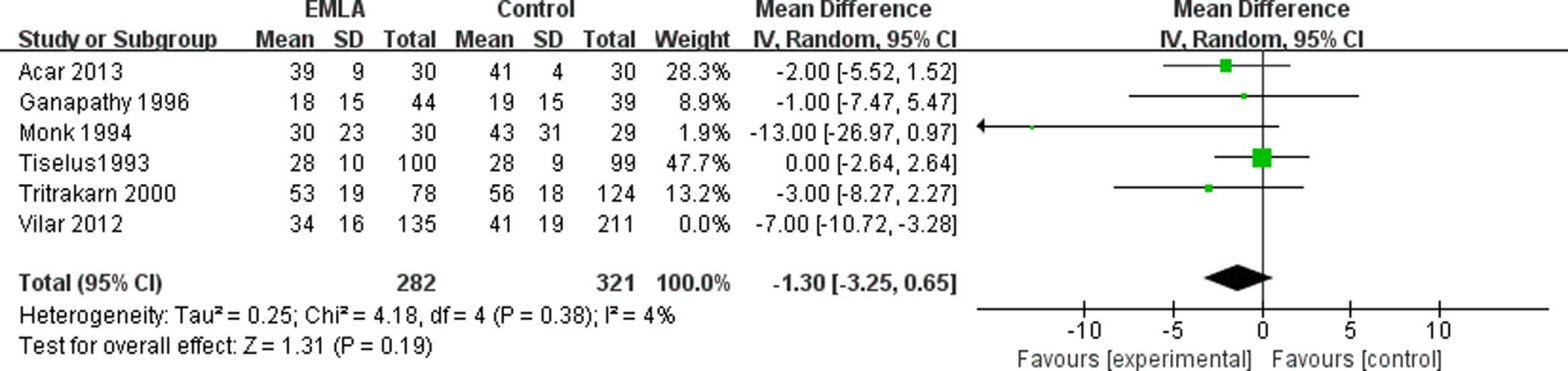
Forest plot for the meta-analysis of pain control after the sensitive analysis.

**Fig 4:**
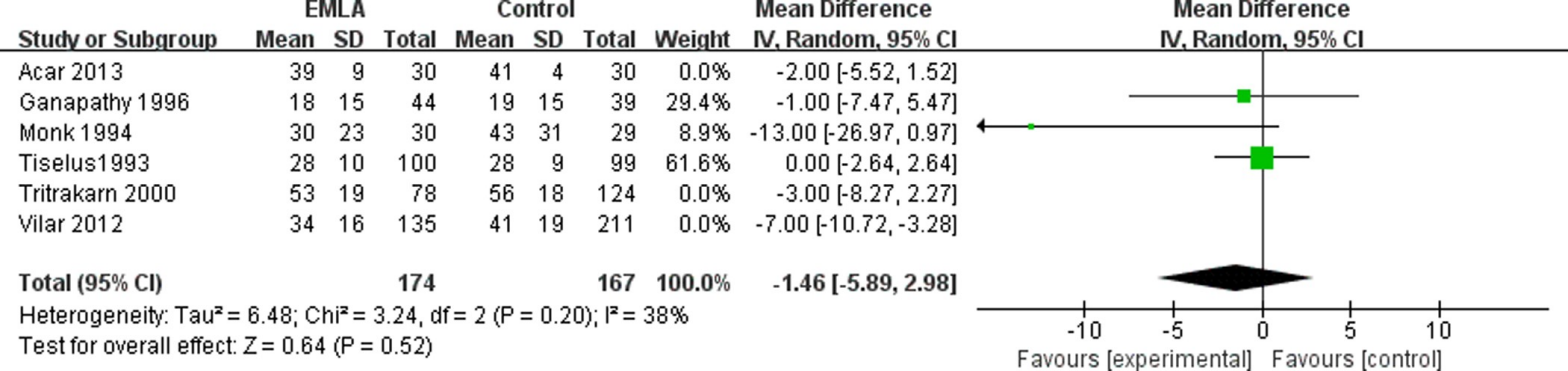
Forest plot for the meta-analysis of pain control with premedication.

**Fig 5:**
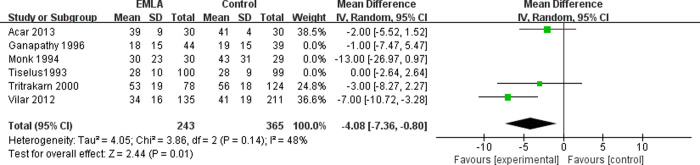
Forest plot for the meta-analysis of pain control without premedication.

The six included RCTs reported the pain level using a visual analogue scale (VAS) at the end of the session (0 = no pain, 10 = maximal possible pain) [18]. We applied a random-effects model for the analysis of this outcome. The results indicated that compared to the control group, EMLA significantly reduced pain (RR = -2.98, 95% CI = -5.82 to -0.13, P = 0.04) with a heterogeneity of I^2^ = 57% (P = 0.04, Fig 2). A sensitivity analysis was performed to evaluate the stability of the results due to a significant heterogeneity in the primary outcome among studies. After removing one study at a time, we found that the heterogeneity was mainly caused by Vilar *et al*. [17]. After removing this study, the pain reduction effect by EMLA became insignificant (RR = -1.30, 95% CI = -3.25 to -0.65, P = 0.19) with a low heterogeneity of I^2^ = 4% (P = 0.38, Fig 3). To further investigate this contradictory result, we performed a subgroup analysis. The studies with analgesics premedication showed that EMLA did not significantly reduce pain (RR = -1.46, 95% CI = -5.89 to 2.98, P = 0.52) with a heterogeneity of I^2^ = 38% (P = 0.52, Fig 4). On the other hand, the studies without premedication showed that the pain relief effect is significant (RR = -4.08, 95% CI = -7.36 to -0.65, P = -0.80) with a heterogeneity of I^2^ = 48% (P = 0.14, Fig 5).

### Analgesic requirement

Four RCTs reported data on analgesic requirement. However, different kinds and dosages of analgesics were provided and the data could not be analysed. Monk *et al*., Ganapathy *et al*., and Acar *et al*. demonstrated that the dosage of analgesics required did not reduce following EMLA [9, 13, 14]. However, Tiselus *et al*. showed that there was a significant reduction in the number of patients who needed additional analgesics and also reported a decreased dosage of analgesics and sedative drugs [15].

## Discussion

EMLA cream is a local anaesthetic and has been proven to effectively relieve pain with few complications in both children and adults since the 1990s. It has a wide range of applications in some outpatient urological and andrological procedures [19]. ESWL is the most accepted and frequently used method for urinary stone in current clinical practice [20]. In addition to the functionality of kidney, stone characteristics, and other factors, patient’s compliance also determines the success of ESWL. Thus, many adjuvant therapies have been investigated to reduce the pain and anxiety throughout the procedure to ultimately improve compliance. Some of these methods have proven to be very effective and have been used in clinical practice such as some anesthetics, analgesics, music therapy and sterile water injection therapy [21–23]. Demir *et al*. showed that decreasing discomfort and reassuring the patient are important for the success of repeated sessions of ESWL [18]. Thus, medication to control anxiety and pain are widely used. Nevertheless, these pharmacotherapies are not only costly but also may lead to side-effects like respiratory depression, hypotension, and allergic reactions [2]. Our systematic review and meta-analysis study investigated whether the application of EMLA reduces the pain of patients undergoing ESWL treatment.

Our result suggested that there was a very limited analgesic effect of EMLA. Interestingly, a study by Tritrakarn and colleagues showed that both EMLA and placebo cream provided anaesthetic effect compared with no intervention [16]. Therefore, the authors proposed that the cream itself was more important than the local cutaneous anaesthetic effect to reduce the pain. An additional explanation could be that a cream-skin interface has less absorption and reflection of energy, leading to less skin trauma and pain. On the other hand, this interface also brings more energy transmission to the stones, which results in a lower numbers of required shock waves and total energy as demonstrated by Vilar *et al*. [17, 24]. Accordingly, the authors found better stone fragmentation in the EMLA-treated group. Ultimately, the analgesic effect of EMLA does not reduce the need for analgesia as shown by most studies in our meta-analysis. Compared to the other studies, there was no additional analgesia during the ESWL procedure in Vilar *et al*. [17]. The authors reported that 12 patients in the EMLA group and 31 patients in the placebo were unable to finish the procedure due to intolerable pain. Monk *et al*. also did not observe significant differences in the reduction of pain and analgesic requirement. However, their sub-analysis of the energy level of the shock waves showed that EMLA was not effective at low energy levels of 10, 12, and 15 kV but did significantly reduce pain at higher energy levels of 18 and 20 kV. Dividing the patient population by gender additionally revealed that EMLA was only effective in males. However, their sample size was likely too small to provide conclusive evidence. Therefore, the authors concluded that EMLA does not reduce the need for analgesics nor does it replace the effects of analgesics [13]. This finding might be because the pain from deep tissue is so pronounced that only reducing superficial tissue pain is clinically insignificant. It is well known that the pain originates from both the skin and the deeper viscera [25]. According to previous studies, cavitation-mediated stimulation of nerve fibres is the main reason for pain during ESWL [4]. EMLA produces better cutaneous analgesia but inadequately suppresses pain in deep tissues. Thus, we do not recommend routine use of EMLA during ESWL, although it may be a choice for patients who are unable to receive other analgesics.

To the best of our knowledge, this is the first systematic review and meta-analysis investigating the impact of EMLA on pain control during ESWL. Several limitations remain in this study. Firstly, the characteristics of stones including stone size, position, composition, and severity of obstruction, which may affect the pain level of the procedure, were not subject to subgroup analysis. Secondly, different ESWL machines cause different pain and noise which also bring the impact on results. Lastly, missing and unpublished data led to bias in the true impact of EMLA.

## Conclusion

In conclusion, the results of this systematic review showed that topical use of EMLA was effective for reducing pain during EWSL. However, this analgesic effect was limited and did not reduce the need for analgesics.

## Supporting information

S1 Checklist Eutectic mixture of local anaesthetics for pain reduction during shockwave lithotripsy: A systematic review and meta-analysis. (DOC)

## References

[1] Amato M, Lusini ML, Nelli F. Epidemiology of nephrolithiasis today. Urol Int. 2004;72 Suppl 1:1–5. Epub 2004/05/11. 10.1159/000076582 . 15133324

[2] Türk C, Petřík A, Sarica K, Seitz C, Skolarikos A, Straub M, et al EAU Guidelines on Interventional Treatment for Urolithiasis. 2016;69(3):475–82. 10.1016/j.eururo.2015.07.041 26344917

[3] Akin Y, Yucel S. Long-term effects of pediatric extracorporeal shockwave lithotripsy on renal function. Research & Reports in Urology 2014;6(1):21–5. 10.2147/RRU.S40965 24892029 PMC4011895

[4] Ozsaker E, Diramali A. The effect of transcutaneous electrical nerve stimulation for pain relief during extracorporeal shock-wave lithotripsy procedure. Pain Manag Nurs. 2014;15(1):59–68. Epub 2014/03/08. 10.1016/j.pmn.2012.06.003 . 24602425

[5] Yilmaz E, Ozcan S, Basar M, Basar H, Batislam E, Ferhat M. Music decreases anxiety and provides sedation in extracorporeal shock wave lithotripsy. Urology. 2003;61(2):282–6. Epub 2003/02/25. 10.1016/s0090-4295(02)02375-0 . 12597931

[6] Takmaz SA, Inan N, Goktug A, Erdogan I, Sunay M, Ceyhan A. The analgesic effect of 8 and 16 mg lornoxicam administered before shock wave lithotripsy: a randomized, double-blind, controlled study. Urology. 2008;72(2):282–5. Epub 2008/05/20. 10.1016/j.urology.2008.03.037 . 18485457

[7] Salinas AS, Lorenzo-Romero J, Segura M, Calero MR, Hernández-Millán I, Martínez-Martín M, et al Factors determining analgesic and sedative drug requirements during extracorporeal shock wave lithotripsy. Urol Int. 1999;63(2):92–101. Epub 1999/12/11. 10.1159/000030425 . 10592496

[8] Gupta NP, Kumar A. Analgesia for pain control during extracorporeal shock wave lithotripsy: Current status. Indian Journal of Urology Iju Journal of the Urological Society of India. 10.4103/0970-1591.40607 2008;24(2):155–8. 19468389 PMC2684259

[9] Acar A, Erhan E, Deniz MN, Ugur G. The Effect of EMLA Cream on Patient-Controlled Analgesia with Remifentanil in ESWL Procedure: A Placebo-Controlled Randomized Study. 10.5812/aapm.7790 Anesthesiology & Pain Medicine. 2013;2(3):119–22. 24244921 PMC3821126

[10] Higgins JP, Thompson SG. Quantifying heterogeneity in a meta-analysis. Stat Med. 2002;21(11):1539–58. Epub 2002/07/12. 10.1002/sim.1186 . 12111919

[11] JADAD A. Assessing the quality of reports of randomized clinical trials: is blinding necessary? Control Clin Trials. 10.1016/0197-2456(95)00134-4 1996;17. 8721797

[12] Kjaergard LL, Villumsen J, Gluud C. Reported Methodologic Quality and Discrepancies between Large and Small Randomized Trials in Meta-Analyse. Annals of Internal Medicine.2001;135(11):982–9. 10.7326/0003-4819-135-11-200112040-00010 11730399

[13] Monk TG, Ding Y, White PF, Albala DM, Clayman RV. Effect of Topical Eutectic Mixture of Local Anesthetics on Pain Response and Analgesic Requirement During Lithotripsy Procedures. Anesthesia & Analgesia. 1994;79(3):506 10.1213/00000539-199409000-00018 8067556

[14] Ganapathy S, Razvi H, Moote C, Parkin J, Yee I, Gverzdys S, et al Eutectic mixture of local anaesthetics is not effective for extracorporeal shock wave lithotripsy. Canadian journal of anaesthesia = Journal canadien d'anesthesie. 1996;43(10):1030–4. Epub 1996/10/01. 10.1007/BF03011905 . 8896855

[15] Tiselius HG. Cutaneous anesthesia with lidocaine-prilocaine cream: a useful adjunct during shock wave lithotripsy with analgesic sedation. J Urol.1993;149(1):8–11. Epub 1993/01/01. 10.1016/s0022-5347(17)35983-9 . 8417220

[16] Tritrakarn T, Lertakyamanee J, Koompong P, Soontrapa S, Somprakit P, Tantiwong A, et al Both EMLA and placebo cream reduced pain during extracorporeal piezoelectric shock wave lithotripsy with the Piezolith 2300. Anesthesiology. 2000;92(4):1049–54. Epub 2001/02/07. 10.1097/00000542-200004000-00023 . 10754625

[17] Gallego Vilar D, García Fadrique G, Di Capua Sacoto C, Beltran Persiva J, Perez Mestre M, De Francia JA, et al Topical EMLA for pain control during extracorporeal shock wave lithotripsy: prospective, comparative, randomized, double-blind study. Urol Res. 2012;40(5):575–9. Epub 2012/05/05. 10.1007/s00240-012-0468-0 . 22555869

[18] Wewers ME, Lowe NK. A Critical Review of Visual Analogue Scales in the Measurement of Clinical Phenomena. Research in Nursing & Health. 1990;13(4):227–36. 10.1002/nur.4770130405 2197679

[19] Buckley MM, Benfield P. Eutectic lidocaine/prilocaine cream. A review of the topical anaesthetic/analgesic efficacy of a eutectic mixture of local anaesthetics (EMLA). 1993;46(1):126–51. 10.2165/00003495-199346010-00008 7691503

[20] Pradère B, Doizi S, Proietti S, Brachlow J, Traxer O. Evaluation of Guidelines for Surgical Management of Urolithiasis. J Urol. 2018;199(5):1267–71. Epub 2017/12/10. 10.1016/j.juro.2017.11.111 . 29221932

[21] Kumar A, Gupta NP, Hemal AK, Wadhwa P. Comparison of three analgesic regimens for pain control during shockwave lithotripsy using Dornier Delta Compact lithotripter: a randomized clinical trial. J Endourol. 2007;21(6):578–82. Epub 2007/07/20. 10.1089/end.2006.0359 . 17638549

[22] Ordaz Jurado DG, Budia Alba A, Bahilo Mateu P, Trassierra Villa M, López-Acón D, Boronat Tormo F. Shockwave lithotripsy with music: Less painful and more satisfactory treatment. Actas urologicas espanolas. 2017;41(9):584–9. Epub 2017/04/17. 10.1016/j.acuro.2017.01.012 . 28412009

[23] Gul A, Gul M. Intracutaneous sterile water injection for pain relief during extracorporeal shock wave lithotripsy: comparison with diclofenac sodium. Urolithiasis. 2020;48(2):103–8. Epub 2019/07/07. 10.1007/s00240-019-01147-9 . 31278470

[24] Cartledge JJ, Cross WR, Lloyd SN, Joyce AD. The efficacy of a range of contact media as coupling agents in extracorporeal shockwave lithotripsy. Bju International. 2001;88(4):321–4. 10.1046/j.1464-410x.2001.02289.x 11564013

[25] Malhotra V, Long CW, Meister MJ. Intercostal blocks with local infiltration anesthesia for extracorporeal shock wave lithotripsy. Anesthesia and analgesia. 1987;66(1):85–8. Epub 1987/01/01. . 3541689

