# Supplementary material for: Eutectic mixture of local anaesthetics for pain reduction during extracorporeal shockwave lithotripsy: A systematic review and meta-analysis
Source: PLoS One. 2020 Oct 5;15(10):e0237783. doi: 10.1371/journal.pone.0237783 (PMC7535034; doi:10.1371/journal.pone.0237783)
Supplement: S1 Checklist — (DOC) [file pone.0237783.s001.doc]

| **Section/topic** | **#** | **Checklist item** | **Reported on page #** |
| --- | --- | --- | --- |
| **TITLE** | | |  |
| Title | 1 | Effect of Eutectic mixture of local anaesthetics on pain reducing During Extracorporeal Shockwave Lithotripsy: A Systematic Review and Meta-Analysis | 1 |
| **ABSTRACT** | | |  |
| Structured summary | 2 | Objective: A systematic review and meta-analysis was conducted to explore effect of Eutectic mixture of local anaesthetics (EMLA) on pain reducing During Extracorporeal Shockwave Lithotripsy (ESWL)  Methods: PubMed, Web of Science, Embase, EBSCO, and Cochrane library databases (updated March 2020) were searched for randomised controlled trials (RCTs) assessing Effect of EMLA for patients underwent ESWL. The search strategy and study selection process were managed according to the PRISMA statement.  Results: Six RCTs were included in the meta-analysis. Overall, The results indicated that compared to the control group, EMLA significantly reduced pain (RR = -2.98, 95% CI = -5.82 to -0.13, P = 0.04) with a heterogeneity of I2 = 57% (P = 0.04).Subgroup analysis shows that EMLA insignificantly reduced pain (RR = -1.46, 95% CI = -5.89 to 2.98, P = 0.52) with heterogeneity of I2 = 38% (P = 0.52) when the patients took an analgesics premedication . On the other hand, the studies without premedication showed the pain relief effect is significant (RR = -4.08, 95% CI = -7.36 to -0.65, P = -0.80) with heterogeneity of I2 = 48% (P = 0.14). And most studies show there in no difference in the analgesic requirement.  Conclusions: EMLA is effective for reducing pain during EWSL, However, this analgesic effect is limited and does not reduce the use of analgesics. | 2 |
| **INTRODUCTION** | | |  |
| Rationale | 3 | To reduce the levels of pain and anxiety and increase the compliance of the patients, various complementary like general anesthesia and drug analgesia were induced into clinical practice. Though these approaches have been proven effective, it is still not highly recommended due to the drug side-effects and its cost. A local anesthetic, EMLA cream, has been confirmed to be effect on pain relief with low complications in both children and adults since 1990s. In some outpatient urological and andrological procedures, it also has a wide range of applications. A few direct compared studies have reported the effect of EMLA on reducing pain and anesthetic requirement on patients during the ESWL. However, the result remained controversial. | 3 |
| Objectives | 4 | We conducted a systematic review and meta-analysis of randomized controlled trials (RCTs) to find out the effect the using of EMLA in the procedure. | 4 |
| **METHODS** | | |  |
| Protocol and registration | 5 | This study is not registered. | - |
| Eligibility criteria | 6 | (1) RCT study design, (2) comparison between ESWL with EMLA versus ESWL with placebo, (3) adequate reporting of data provided for analysis, and (4) full text in English. | 4-5 |
| Information sources | 7 | We systematically searched several databases including PubMed, Embase, Web of Science, EBSCO, and the Cochrane library from up to March 2020 with the following keywords: “topical anaesthetics”, “eutectic mixture of local anaesthetics”, “pain” and “shock wave lithotripsy”. The list of retrieved studies and relevant reviews were assessed manually, and the process mentioned above was performed several times to ensure that all eligible studies were included. | 5 |
| Search | 8 | The article selection process was performed in accordance with the PRISMA guidelines. | 5 |
| Study selection | 9 | The reference lists of retrieved studies and relevant reviews were hand-searched, and the process mentioned above was repeatedly performed for ensuring that all eligible studies were included. | 5 |
| Data collection process | 10 | Data were independently extracted by two investigators. Discrepancies were resolved by consensus. | 5 |
| Data items | 11 | We assess Standard Mean differences (Std. MDs) with 95% confidence intervals (CIs) for continuous outcomes, and risk ratios (RR) with 95% CIs for dichotomous outcomes. Heterogeneity is evaluated using the I^2^ statistic, and I2 > 50% indicates significant heterogeneity. | 5 |
| Risk of bias in individual studies | 12 | We used the Jadad Scale to evaluate the quality of the study. | - |
| Summary measures | 13 | Risk ratios (RR) with 95% confidence intervals (CIs) were calculated for dichotomous outcomes. Heterogeneity was evaluated using the I2 statistic | 5 |
| Synthesis of results | 14 | I2 > 50% taken to indicate significant heterogeneity. | 5 |

Page 1 of 2

| **Section/topic** | **#** | **Checklist item** | **Reported on page #** |
| --- | --- | --- | --- |
| Risk of bias across studies | 15 | The Jadad scores of the included studies varied from two to four. One study was considered to be low quality while other three studies were considered to be high quality. | - |
| Additional analyses | 16 | Sensitivity analysis was performed for evaluating the influence of a single study on the overall estimate by omitting one study in turn or performing subgroup analysis. | 5 |
| **RESULTS** | | |  |
| Study selection | 17 | In total, 162 articles were initially identified from the databases. After removing duplicates, 101 articles were retained. Then 92 studies were excluded from our study due to unrelated abstracts and titles. We also excluded from our analysis: one article for its study design (not RCT), one articles for insufficient data and one article for inconformity of outcomes. Finally, six RCTs that satisfied the inclusion criteria were enrolled in this meta-analysis | 6 |
| Study characteristics | 18 | Shown in the table 1. | 7 |
| Risk of bias within studies | 19 | - | - |
| Results of individual studies | 20 | Shown in the table 1. | 7 |
| Synthesis of results | 21 | We applied a random-effects model for the analysis of this outcome. The results indicated that compared to the control group, EMLA significantly reduced pain (RR = -2.98, 95% CI = -5.82 to -0.13, P = 0.04) with a heterogeneity of I2 = 57% (P = 0.04). | 7-8 |
| Risk of bias across studies | 22 | - | - |
| Additional analysis | 23 | A sensitivity analysis was performed to evaluate the stability of the results due to a significant heterogeneity in the outcome of pain control among studies. After removing one study at a time, we found that the heterogeneity was mainly a result of data from Vilar et al. However, after removing this study, the result became insignificantly (RR = -1.30, 95% CI = -3.25 to -0.65, P = 0.19) with a low heterogeneity of I2 = 4% (P = 0.38, Figure 3). This contradictory conclusion is very puzzling. Thus, a subgroup analysis was made. The studies with analgesics premedication showed EMLA insignificantly reduced pain (RR = -1.46, 95% CI = -5.89 to 2.98, P = 0.52) with heterogeneity of I2 = 38% (P = 0.52). On the other hand, the studies without premedication showed the pain relief effect is significant (RR = -4.08, 95% CI = -7.36 to -0.65, P = -0.80) with heterogeneity of I2 = 48% (P = 0.14) | 8 |
| **DISCUSSION** | | |  |
| Summary of evidence | 24 | The topical use EMLA is effective for reducing pain during EWSL, However, this analgesic effect is limited and does not reduce the use of analgesics | 9 |
| Limitations | 25 | Firstly, the characteristics of stone including stone size, position, composition, and severity of obstruction which may affect the pain of the procedure could not make subgroup to analysis. Secondly, more types of intraoperative analgesics is worth pondering in future study. Lastly, missing and unpublished data also led to bias in the true impact of EMLA. | 10 |
| Conclusions | 26 | In conclusion, the results of this systematic review showed that topical use EMLA is effective for reducing pain during EWSL, However, this analgesic effect is limited and does not reduce the use of analgesics | 10 |
| **FUNDING** | | |  |
| Funding | 27 | N/A | - |

*From:*  Moher D, Liberati A, Tetzlaff J, Altman DG, The PRISMA Group (2009). Preferred Reporting Items for Systematic Reviews and Meta-Analyses: The PRISMA Statement. PLoS Med 6(7): e1000097. doi:10.1371/journal.pmed1000097

For more information, visit: **www.prisma-statement.org**.

Page 2 of 2
